# Accelerometry-Based Metrics to Evaluate the Relative Use of the More Affected Arm during Daily Activities in Adults Living with Cerebral Palsy

**DOI:** 10.3390/s22031022

**Published:** 2022-01-28

**Authors:** Isabelle Poitras, Jade Clouâtre, Alexandre Campeau-Lecours, Catherine Mercier

**Affiliations:** 1 Centre for Interdisciplinary Research in Rehabilitation and Social Integration, Centre Intégré Universitaire de Santé et Services Sociaux de la Capitale-Nationale, Quebec City, QC G1M 2S8, Canada; Isabelle.poitras.2@ulaval.ca (I.P.); jade.clouatre.1@ulaval.ca (J.C.); alexandre.campeau-lecours@gmc.ulaval.ca (A.C.-L.); 2 Department of Rehabilitation, Laval University, Quebec City, QC G1V 0A6, Canada; 3 Department of Mechanical Engineering, Laval University, Quebec City, QC G1V 0A6, Canada

**Keywords:** cerebral palsy, accelerometry, upper limb, bimanual function, unimanual function, psychometric properties

## Abstract

Adults living with cerebral palsy (CP) report bimanual and unimanual difficulties that interfere with their participation in activities of daily living (ADL). There is a lack of quantitative methods to assess the impact of these motor dysfunctions on the relative use of each arm. The objective of this study was to evaluate the concurrent and discriminative validity of accelerometry-based metrics when used to assess bimanual and unimanual functions. Methods: A group of control subjects and hemiplegic adults living with CP performed six ADL tasks, during which they were wearing an Actigraph GT9X on each wrist and being filmed. Four bimanual and unimanual metrics were calculated from both accelerometry-based and video-based data; these metrics were then compared to one other with an intraclass correlation coefficient (ICC). Some of these metrics were previously validated in other clinical population, while others were novel. The discriminative validity was assessed through comparisons between groups and between tasks. Results: The concurrent validity was considered as good to excellent (ICC = 0.61–0.97) depending on the experience of the raters. The tasks made it possible to discriminate between groups. Conclusion: The proposed accelerometry-based metrics are a promising tool to evaluate bimanual and unimanual functions in adults living with CP.

## 1. Introduction

The most predominant type of cerebral palsy (CP) is spastic hemiplegia [1] in which greater sensorimotor deficits are reported in the side of the body contralateral to the cerebral lesion [2,3] and weaker deficits are reported in the ipsilateral side [4]. Besides these unilateral motor deficits, there is a lack of bimanual coordination [5] that has a major impact on participation and independence in activities of daily living (ADL), since the majority of ADL require a coordinated use of both hands (e.g., to fold a towel) [6,7]. However, the assessment of bimanual functions and of the relative use of each arm remain challenging: most assessment tools are based on either a person’s own judgement about their motor performance (e.g., ABIlHAND-Kids questionnaire [8]), on the extrapolation of unimanual functions of the more affected hand (e.g., Box and Blocks [9]), or on clinical observation (e.g., Assisting Hand Assessment [10]). Each of these methods has limitations. Subjective assessments (i.e., questionnaires) provide relevant clinical information but are subject to multiple biases, such as recall bias [11] and desire for social approval [12]. Furthermore, it is known that extrapolating bimanual functions purely by assessing unimanual functions underestimates deficits in bimanual functions, since the impact of deficits in the less affected hand and the independent mechanisms underlying bimanual functions are disregarded in the evaluation [13]. Finally, clinical observations are influenced by therapist bias [14] and observer bias [15], besides being subject to an expected variability across different evaluators. Taken together, these points highlight the need for a quantitative and objective assessment of bimanual functions.

Accelerometers have been suggested to quantify movements of each arm and, by extension, the relative use of each arm during ADL. These tools, when integrated in portable casings, have the advantage of being portable, light, easy to use [16], and well-accepted when carried at the wrist [17,18] and are a technology already integrated into smartwatches and smartphones. Different metrics have been developed to quantify the use of upper extremities in different tasks and contexts; most of these metrics report the total amount of time a given arm is in motion relative to the other (*use ratio* (*UR*) [19]) or the intensity of movement of a given arm relative to the other (asymmetry index [20,21]). These metrics provide useful information about the total activity of each arm during a certain period of time while reducing the impact of arm swing [22] on the estimation of purposeful activity. However, these metrics tend to ignore the level of interaction between both arms—i.e., whether they act simultaneously (bimanual function) or sequentially (unimanual function). Recent studies have addressed this problem by proposing other accelerometry-based metrics (e.g., mono-arm use index (MAUI) and bilateral-arm use index (BAUI)) that separate data for the simultaneous use of both arms from data for the isolated use of one arm [6,23,24,25]. The validity of these metrics has been assessed in healthy subjects [23], stroke survivors [6,26] and children living with CP [24,27], but no study has yet been conducted in adults living with CP. While adults with CP are often compared to stroke survivors, important differences must be considered: Since CP is a congenital disorder (or resulting from an insult occurring early in life), the less affected arm is generally the dominant arm; in stroke, on the other hand, either the dominant or the non-dominant arm can be affected, since stroke is an acquired brain injury. Additionally, comparing adults with CP to children with CP can be misleading, since it has been suggested that everyday movements and use of the arms differ greatly between adults and children [28]. Literature evaluating adults living with CP is scarce, which restricts the number of valid assessment tools available for clinicians all the more. This is a major concern that needs to be addressed, particularly since the motor functions of adults living with CP tend to decline with aging [29,30].

Prior to the implantation of accelerometry-based metrics for clinical use in a given clinical population, it is essential to assess the psychometric properties of these metrics. Two important psychometric properties are concurrent and discriminative validity. Concurrent validity refers to measuring how well a new assessment method compares to a well-established one. To establish the concurrent validity of accelerometry-based metrics, they are generally compared to clinician observations (performed live or through video analyses) [31,32,33]. The discriminative validity refers to the capacity of an assessment method to be able to distinguish between individuals. For accelerometry-based metrics, this is generally established by testing whether metrics allow to distinguish between a group of healthy controls and a clinical population of interest or between different levels of impairment in individuals from that clinical population [34,35]. 

The objective of this study was to evaluate the concurrent and discriminative validity of accelerometry-based metrics that aim to characterize bimanual and unimanual functions in adults living with cerebral palsy. Because concurrent validity was tested against a video-based method, a secondary objective was to assess the inter-rater reliability of this video-based method. 

## 2. Materials and Methods

### 2.1. Participants

The participants were recruited through the Université Laval mailing list and the health records of the Centre intégré universitaire de santé et de services sociaux de la Capitale-Nationale (CIUSSS-CN). A minimal sample size of 14 participants (seven by group) was set (sample size required to respect: α = 0.05, β = 0.2, and an expected ICC = 0.85) [36]. The inclusion criteria for the CTRL group were: (1) being aged between 18 and 65 years of age; and (2) reporting no musculoskeletal pain or neurological disease that interferes with task execution. The inclusion criteria for the CP group were: (1) being aged between 18 and 65 years of age; (2) having a diagnosis of hemiplegic cerebral palsy; and (3) having a level of I, II, or III on the Manual Ability Classification System (MACS) [37]. The project was approved by the local ethic committee (Ethics #2018-609, CIUSSS-CN) and all participants provided their written informed consent prior to the experiment.

### 2.2. Experimental Setup and Tasks

The participants took part in a one-session assessment, in which they were requested to perform six tasks that required the use of both arms and that were representative of ADL (see Table 1 for more details). The different tasks were selected to cover a heterogeneous level of bimanual use as some are impossible to perform correctly (e.g., folding towels) with one arm while other are possible to perform with one arm (e.g., pouring a glass of water), allowing participants to use the more comfortable strategy for them. The participants wore an Actigraph GT9X Link on each wrist (sample rate = 100 Hz, internal memory of 4Go, accelerometer dynamic range of ±8 g, Actigraph LLC, Pensacola, FL, USA) while being filmed (sample rate of 30 frames/second). They were asked to perform specific shoulder flexion movements at the beginning (two repetitions) and at the end (three repetitions) of each task, to synchronize video and acceleration data and to generate data epochs. 

### 2.3. Video Processing

The video data were processed using custom Python software (Python Software Foundation, Wilmington, DE, USA. Python Language Reference, version 3.7) that makes it possible to slow video playback down to 25% of its original speed. To assess inter-rater reliability, two evaluators performed the rating individually (rater 1 (experienced) = certified occupational therapist with 5 years of experience, rater 2 (naive) = student in occupational therapy). Both evaluators were members of the research team. They were instructed that for the purpose of the study ‘’arm movement’’ was defined as any upper extremity movement requiring a displacement of the wrist visually detectable. They first watched the video at its original speed, and then at 25% of the original speed, for each arm. At the 25% speed, both evaluators were tasked to identify epochs in which arm movement was present by pressing the space bar (allocating a value of 1 was allocated when the space bar was pressed (arm movement) and a 0 was allocated if not (no arm movement)). The ratings obtained from the video processing were interpolated to obtain data equivalent to 100 frames/second, which allowed for a comparison with accelerometry data. 

### 2.4. Accelerometry Processing

The pre-processing of the accelerometry data was performed using ActiLife 6 software (Actigraph LLC, Pensacola, FL, USA) to initially obtain the raw data. The data acquisition, filtering (continuous eighth order bandpass filter) and transformation to activity counts is performed at 100 Hz and then summed into epochs of 1 s; see [38] for details. The filtering of the data and activity count calculations were performed in customized MATLAB software (1. MATLAB version 9.6.0 (R2019a). Natick, MA, USA: The MathWorks Inc.; 2010). The vector magnitude ($VM_{i}=\sqrt{s_{x_{i}}{}^{2}+s_{y_{i}}{}^{2}+s_{z_{i}}{}^{2}})\;$ that represents the norm of the activity counts on the three axes *x*, *y* and *z* (where $sx_{i},$ $sy_{i}$ and $sz_{i}$ represent the sum of the activity counts for a specific epoch [i]), was first calculated for each arm. A *VM* of more than 100 was considered as indicating the presence of arm movement (allocating a value of 1 to this epoch) and a *VM* of less than 100 was considered to indicate absence of movement (allocating a value of 0 to this epoch). The *VM* threshold of 100 was set in a prior pilot study by comparing a variety of movements such as large and small movements, arm swing while walking, tremors and acceleration values while driving a car and taking the bus. It was decided to a threshold set a priori (rather than adjusting the threshold based on video-based rating of the current data) to avoid overestimating concurrent validity, which would decrease the possibility to generalize the results to a variety of tasks. This allowed for a comparison with video data.

### 2.5. Metrics Calculation

The metrics are classified in two categories: bimanual and unimanual. The bimanual metric shows *the percentage of bimanual use*. The three unimanual metrics are the *use ratio* (*UR*), the *percentage of dominant use*, and the *percentage of non-dominant use*. For each formula, “*A*”, which is either 0 or 1, represents the value of activity given by either the accelerometer or the video rater.

#### 2.5.1. Bimanual Metrics

The *percentage of bimanual use* represents the proportion of epochs during which both arms are moving simultaneously, versus the total amount of epochs where at least one arm is moving.

(1) $$percentage\;of\;bimanual\;use=\frac{\Sigma (A_{non\text{-}dom}\neq 0\;\mathrm{AND}\;A_{dom}\neq 0)\;}{\Sigma A_{\mathrm{total}}}\times 100$$

where *A_(non-dom)_* ≠ 0 represents epochs where the non-dominant arm is moving, *A_dom_* ≠ 0 represents epochs where the dominant arm is moving and the “AND” indicates that both are true simultaneously for a given epoch, and therefore that the arms move simultaneously at that given epoch. 

#### 2.5.2. Unimanual Metrics

The *percentage of dominant use* represents the proportion of epochs during which the dominant arm is moving while the non-dominant arm is not moving, versus the total amount of epochs where at least one arm is moving.

(2) $$percentage\;of\;dominant\;use=\frac{\Sigma A_{dom}\;\mathrm{if}\;A_{non\text{-}dom}=0}{\Sigma A_{total}\;}\times 100\;$$

where $A_{non\text{-}dom}=0$ represents the number of epochs where the non-dominant arm is not moving and $A_{total}$ represents the number of epochs where at least one arm is moving.

The *percentage of non-dominant use* represents the proportion of epochs during which the non-dominant arm is moving while the dominant arm is not moving, versus the total amount of epochs where at least one arm is moving.

(3) $$percentage\;of\;non\text{-}dominant\;use=\frac{\Sigma A_{non\text{-}dom}\;\mathrm{if}\;A_{dom}=0}{\Sigma A_{total}}\times 100$$

where $A_{dom}=0$ represents the number of epochs where the dominant arm is not moving and $A_{total}$ represents the number of epochs where at least one arm is moving.

The *UR* represents a ratio of the number of epochs during which the non-dominant arm is used versus the number of epochs the dominant arm is used (*UR* < 1 = more use of the dominant arm, *UR* > 1 = more use of the non-dominant arm, *UR* of 1 = equal use of both arms).

(4) $$UR=\frac{movement\;duration\;non\text{-}dom\;\left(Anon\text{-}dom\;\neq 0\right)}{movement\;duration\;dom\;\left(Adom\neq 0\right)}$$

where $A_{non\text{-}dom}\neq 0$ represents the number of epochs where the non-dominant arm is moving, $A_{dom}\neq 0$ represents the number of epochs where the dominant arm is moving.

These three unimanual metrics are complementary: the *UR* represents the proportion of epochs each limb is used over the whole task, while the *percentage of dominant and non-dominant use* represent the amount of unimanual use, exclusively, over the total period of arm activity. For example, the equal use of both arms in succession and the simultaneous use of the two arms would both result in a *UR* of 1. However, the equal use of both arms in succession would result in a dominant (or non-dominant) use of 50% and the simultaneous use of the two arms would result in a use of 0%.

Note that the sum of the *percentage of dominant use*, *non-dominant use*, and bimanual use is necessarily 100%. 

Also note that the *UR* combines the value of the other metrics into an integrative ratio.

(5) $$UR=\frac{percentage\;of\;bimanual\;use+percentage\;of\;non\;dominant\;use}{percentage\;bimanual\;use+percentage\;of\;dominant\;use}$$

### 2.6. Statistical Analysis

The statistical analyses were performed using the SPSS Statistics for Windows, version 27, and the R Studio software version (2021.09.0 Build 351). The concurrent validity of each metric was assessed by calculating an intraclass correlation coefficient (ICC) between each accelerometry-based metric and its corresponding video-based metric (for rater 1 and 2, separately). In relation to the secondary objective (inter-rater reliability of video-based metrics), ICCs were also calculated between results obtained by rater 1 and rater 2 for each of the four metrics. An ICC below 0.5 indicates a poor correlation, an ICC between 0.5 and 0.75 indicates a moderate correlation, an ICC between 0.76 and 0.9 indicates a good correlation, and an ICC greater than 0.9 indicates an excellent correlation [39]. The confidence intervals on ICCs were also calculated. 

The discriminative validity was calculated by using a rankFD function [40], which is a non-parametric statistical method that is practical in testing whether there was a main effect of the Group, a main effect of the Task, or a “Group X Task interaction” effect, particularly when dealing with small sample sizes. The type of statistic used was the ANOVA Type Statistic (ATS) and their values are reported in the results. The analyses with the rankFD function were separated in three steps, and only significant results were retained for further steps. Step 1 was to identify which metric allow to discriminate between both groups. Step 2 was to determine which task allow to discriminate between groups for each metrics. Step 3 was to identify which tasks are different from one another by comparing them to each other. A correction of Benjamini [41] was applied to all significant results, to diminish multiple analyses bias. Because of the limited sample size, this correction is highly conservative, and uncorrected results are therefore presented in the text. However, both uncorrected and corrected results are presented to allow readers to select the information deemed to be the most appropriate to their needs. 

For all analyses, the statistical significance was set at *p*-value ≤ 0.05.

## 3. Results

### 3.1. Participant Description

The CTRL and the CP groups were composed of eleven participants each. A description of the participants is presented in the Table 2.

### 3.2. Metrics Description

**Figure 1:**
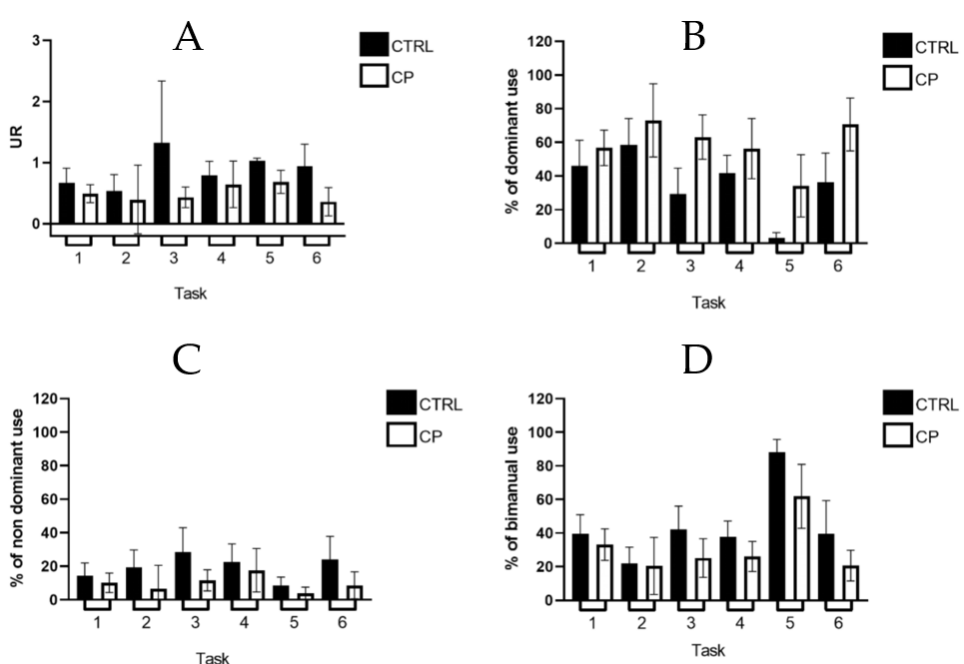
Mean and 95% confidence intervals of each metric by group and task. (**A**): *Use ratio (UR)*. (**B**): *percentage of dominant use*; (**C**): *percentage of non-dominant use*; (**D**): *percentage of bimanual use*; Legend: CTRL = control group; CP = cerebral palsy group.

Figure 1 presents descriptive results for each metric by group and task. This graphical representation shows the varied level of bimanual vs. unimanual functions associated to different tasks. For example, Task 5 shows the highest *percentage of bimanual use* (mean (95% CI)): (CTRL: 88.2% (80.6–95.8); CP: 61.9% (42.9 to 80.9)) while Task 2 shows the lowest (CTRL: 22.16% (12.7 to 31.6); CP: 20.5% (3.5 to 37.5)). The CP group shows a lower level of non-dominant use when compared with the CTRL group for all the tasks and specifically for Task 2 (CTRL: 28.5% (13.8 to 43.1); CP: 6.5% (−7.7 to 20.6)). 6: CTRL: 24.2% (10.5 to 37.9); CP: 8.7% (0.6 to 16.8)). The CP group also shows a lower level of bimanual use (CTRL = 22.16 to 88.19%; CP = 20.47 to 61.9%), for all tasks and more notably for Task 3 (CTRL: 42.2% (28.4 to 56.0); CP: 25.2% (13.7 to 36.8)), 4 (CTRL: 37.7% ( 8.3 to 47.1); CP: 26.1% (17.1 to 35.1)), 5 (CTRL: 88.2% (80.6 to 95.8); CP: 61.9% (42.9 to 80.9)) and 6 (CTRL: 39.7% (20.0 to 59.4); CP: 20.7% (11.6 to 29.8)). Statistical analyses related to these quantitative observations are reported in the Discriminative Validity section.

### 3.3. Concurrent Validity and Inter-Rater Reliability

**Figure 2:**
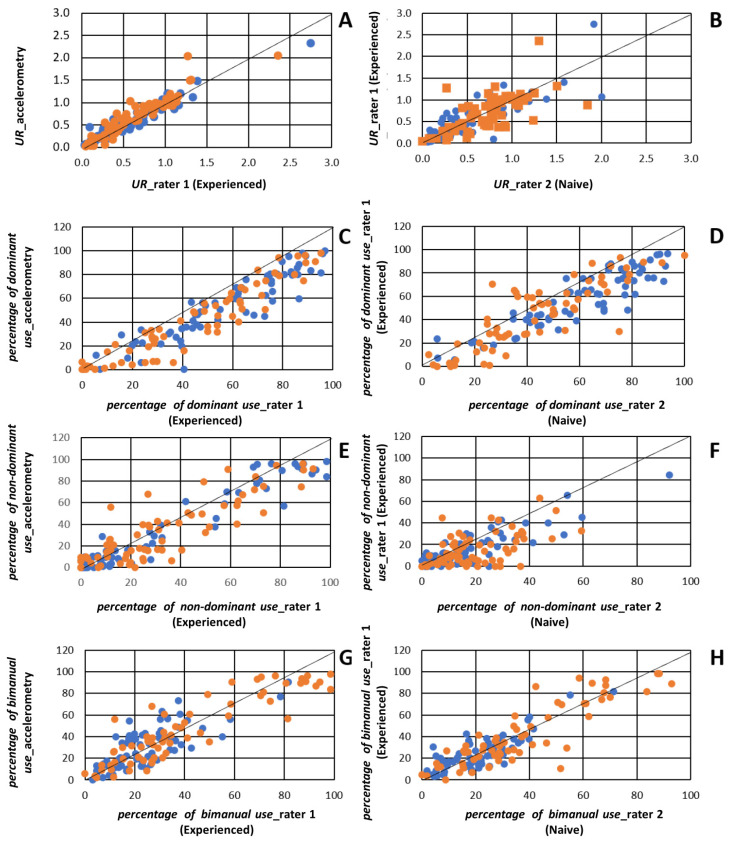
Graphical representation of association reported in Table 3: (**A**): Association between video-based *use ratio (UR)* Experienced; (**B**): Association between video-based *UR* of both raters; (**C**): Association between video-based and accelerometry-based *percentage of dominant use*; (**D**): Association between video-based *percentage of dominant use* of both raters; (**E**): Association between video-based and accelerometry-based *% of non-dominant use*; (**F**): Association between video-based *percentage of non-dominant use* of both raters); (**G**): Association between video-based and accelerometry-based *percentage of bimanual use*; (**H**): Association between *percentage of bimanual use* of both raters. Note that when data from a single rater is presented on a graph, data from rater 1 (experienced) is depicted, Legend: Blue dots= control group; Orange dots = cerebral palsy group.

Table 3 presents the results for the concurrent validity of accelerometry and video-based metrics for all tasks and all subjects taken together, as well as results for the inter-rater reliability of video-based metrics. The concurrent validity is considered as good to excellent (mean (95% CI): *UR*: 0.97 (0.96 to 0.98); *percentage of dominant use*: 0.92 (0.88 to 0.95); *percentage of non-dominant use*: 0.88 (0.84 to 0.92); *percentage of bimanual use*: 0.85 (0.75 to 0.91); all *p*-values < 0.001) when comparing data measured by accelerometry and data calculated by Rater 1 (the experienced therapist). The concurrent validity is considered to be moderate to good (*UR*: 0.74 (0.66 to 0.81); *percentage of dominant use*: 0.78 (0.69 to 0.84); *percentage of non-dominant use*: 0.61 (0.47 to 0.71); *percentage of bimanual use*: 0.65 (0.44 to 0.78), all *p*-values < 0.001) when comparing data measured by accelerometry and data calculated by Rater 2 (the naive evaluator). Inter-rater reliability is considered to be moderate to good (*UR*: 0.69 (0.66 to 0.72); *percentage of dominant use*: 0.87 (0.82 to 0.91); *percentage of non-dominant use*: 0.70 (0.60 to 0.78); *percentage of bimanual use*: 0.85 (0.78 to 0.90; all *p*-values < 0.001). Figure 2 provides a graphical representation of these associations and shows the distribution of each variable across each group. All outcomes show a narrow confidence interval, meaning that results are reliable. 

### 3.4. Discriminative Validity

Table 4 shows the results of the rankFD analyses. These analyses revealed a main effect of the Group and Task for all four metrics (*p* < 0.05), supporting the discriminative validity of these metrics.

Post-hoc analyses were performed to identify which specific tasks allowed the best discrimination between groups. Depending on the specific metrics, between two and four tasks revealed a significant difference between groups: Tasks 3 and 5 were found to be different across groups for all metrics, and Tasks 2 and 6 differed across groups for either one or two metrics, respectively. Tasks 3, 5, and 6 were found to discriminate between groups for *UR* and *percentage of dominant use*; Tasks 2, 3, 5, and 6 were found to discriminate between groups for *percentage of non-dominant use*, and Tasks 3 and 5 were found to discriminate between groups for the *percentage of bimanual use*. The study also assessed whether tasks that successfully discriminated between groups were different from one another (i.e., if they conveyed different information about upper limb use). For three metrics, significant differences were found between Tasks 3 and 5, as well as between Tasks 5 and 6; a single metric differed between Tasks 2 and 3. To summarize, Task 3 (setting the table), Task 5 (folding towels), and Task 6 (putting toothpaste on a toothbrush) are the ones that show the greatest discrimination between groups and tasks.

## 4. Discussion

The results of this study support the validity of the four accelerometry-based metrics proposed to assess the relative use of each upper limb during ADL in adults living with CP. To the best of our knowledge, this is the first study to focus on the validity of such metrics in adults living with CP. The results show a good to excellent convergence with video-based assessments performed by an experienced occupational therapist (video-assessment by a therapist being much more time-consuming). Selected metrics also showed it is possible to discriminate between the groups (CTRL vs. CP) and the tasks performed, with some tasks being mainly unimanual (e.g., putting toothpaste on a toothbrush) and others being mainly bimanual (e.g., folding towels).

Some previous studies have focused on the validity of accelerometry-based metrics in individuals living with CP, but in adults, the validity of accelerometry-based metrics to quantify physical activity has been found to be only moderate to good [42,43]. The use of accelerometry-based metrics in adults with CP therefore appears to be valid for the quantification of diverse motor functions (specifically in measuring upper limb use vs. general physical activity). In children living with CP, a good agreement has been found between video-based and accelerometry-based measurements in measuring arm movement (ratio of use of each arm, without distinguishing between unimanual or bimanual use) over 20 min of free play in a seated position [31]; our results during ADL are similar to the ones reported. This supports the validity of accelerometry-based metrics for measuring upper limb use across age groups (children vs. adults) and across different types of tasks (play in a seated position vs. ADL involving displacements). Other studies support the validity of accelerometry-based metrics to assess the intensity of movement without having focused specifically on upper limb use [6,23] or have showed association between such metrics and clinical assessments [44,45]. Our study expands these findings, by validating both bimanual and unimanual metrics in order to fully characterize the relative use of the more affected arm in relation to the less affected arm.

Moreover, our study has identified three tasks that show good discrimination between groups, and that differ from each other in terms of bimanual vs. unimanual involvement for a rapid evaluation of bimanual and unimanual functions in adults living with CP. This is clinically relevant, since time is often limited in clinical contexts, particularly in acute-care facilities [46]. The three selected tasks give a quick overview of the bimanual and unimanual functions but take only 5 min to perform. Furthermore, since accelerometers are portable and light, they can be used either in clinical or home settings. This makes possible assessments that are performed over a more extensive period of time and in a more ecological environment, which is something that simply cannot be achieved through observation-based assessments performed by a clinician. It should also be noted that accelerometry-based metrics were found to be more closely associated to video-based metrics obtained by an experienced rater, as compared with metrics obtained by a naïve rater. This highlights the plus-value of using accelerometry not only to fully assess the motor capacities, but also as a tool to complete the observations of less-experienced clinicians and non-clinicians.

### 4.1. Study Limitations

This study does have five limitations that should be acknowledged. First, the data collection was limited to a short period of time (approximately 10 min) and to the completion of discrete tasks that are not fully representative of everyday life. That said, the selected tasks provide a practical stand-in for tasks that range from mainly unimanual (e.g., putting toothpaste on a toothbrush) to mainly bimanual (e.g., folding towels). Second, when validating the metrics, the intensity at which a task was performed was not considered: while metrics focusing on intensity of movement rather than on movement time are relevant from a clinical standpoint, the intensity of arm movements during an ADL task cannot be assessed precisely using a video-based method for validation purposes. Third, this study assessed the validity of accelerometry-based measurements for upper limb quantification in a controlled environment and in a specific set of tasks. The validity of these metrics in a real-world environment and for measurement performed over an extended period of time as well as their reliability remain to be established. Fourth, the small number of participants included in this study could theoretically add some bias, but the statistical analysis (using rankFD) and the heterogeneity of motor deficits observed in the CP group (MACS level equally distributed) reduce the impact of potential bias on conclusions. Finally, the fact that the two evaluators were a part of the research team is a limitation as they were aware of the purpose of the study. However, the two observers were naïve to the accelerometry data during video rating, thus reducing the risk of bias.

### 4.2. Clinical Implications

The accelerometry-based metrics validated in this study could be used in initial assessments and follow-up of adults living with CP. More studies will be required to assess the metrics’ reliability and sensitivity to change following a specific intervention. Accelerometry-based metrics would make it possible to assess the progression of motor impairments with aging in adults living with CP, an important question that has yet received limited attention in clinical research. In future studies, it would be interesting to assess to what extent the impairments in the more affected limb, but also in the less affected limb, impact the relative use of each limb, as well as the bimanual use [47].

## 5. Conclusions

The validation of the use of accelerometry-based metrics for assessing bimanual and unimanual functions in adults living with CP provides clinicians and researchers with an interesting tool to evaluate the relative use of each arm during ADL tasks. The proposed metrics make possible a more detailed and quantitative overview of the actual use of the more affected arm and provide more detailed information about bimanual performance. Further studies should focus on the implementation of these metrics during rehabilitation programs, and the use of different metrics based on the movement intensity characteristic in this understudied population. Complementary metrics could also be used to address the quality of the movements performed to provide a full overview of motor performance in adults living with CP [48].

## Figures and Tables

**Table 1:** Description of the six tasks performed by participants.

Task	Description
Cleaning the table	Turning on a tap, filling a bowl of water, turning off the tap, wringing out a towel, and cleaning the table.
Making coffee	Opening a container, picking up a spoon, putting two spoonfuls of coffee in a cup, and closing the jar.
Setting the table	Setting a table for two, with two forks, two knives, two plates, and two glasses.
Pouring a glass of water	Turning on the tap, filling up a pitcher, turning off the tap, and pouring a glass of water.
Folding towels	Folding two large towels and stacking them on top of each other.
Putting toothpaste on a toothbrush	Opening a tube of toothpaste, putting a small amount of toothpaste on a toothbrush, putting the cap back on the toothpaste, and putting both objects on the table.

**Table 2:** Descriptive characteristics of the participants by group.

	CTRL (*n* = 11)	CP (*n* = 11)
Age (mean ± SD years)	27.8 ± 6.6	35.9 ± 13.3
Female	8 (73%)	7 (64%)
Right-handed	10 (91%)	4 (36%)
Side of hemiplegia	-	8 (73%)
MACS levels	-	I = 4 (36.5%) II = 4 (36.5%) III = 3 (27%)

Legend: CP = cerebral palsy group; CTRL = control group; SD = standard deviation; MACS = Manual Ability Classification System.

**Table 3:** Concurrent validity and inter-rater reliability of accelerometry and video-based metrics (Intraclass correlation coefficient, 95% Confidence interval and *p*-value).

Metric	Comparison	ICC	95% Confidence Interval	*p*-Value
*UR*	Accelerometry vs. Rater 1 (Experienced)	0.97	0.96–0.98	<0.001
	Accelerometry vs. Rater 2 (Naive)	0.74	0.66–0.81	<0.001
	Rater 1 (Experienced) vs. Rater 2 (Naïve)	0.69	0.66–0.72	<0.001
*percentage of dominant use*	Accelerometry vs. Rater 1 (Experienced)	0.92	0.88–0.95	<0.001
	Accelerometry vs. Rater 2 (Naive)	0.78	0.69–0.84	<0.001
	Rater 1 vs. Rater 2	0.87	0.82–0.91	<0.001
*percentage non dominant use*	Accelerometry vs. Rater 1 (Experienced)	0.88	0.84–0.92	<0.001
	Accelerometry vs. Rater 2 (Naive)	0.61	0.47–0.71	<0.001
	Rater 1 (Experienced) vs. Rater 2 (Naive)	0.70	0.60–0.78	<0.001
*percentage bimanual use*	Accelerometry vs. Rater 1 (Experienced)	0.85	0.75–0.91	<0.001
	Accelerometry vs. Rater 2 (Naive)	0.65	0.44–0.78	<0.001
	Rater 1 vs. Rater 2	0.85	0.78–0.90	<0.001

Legend: *UR* = *use ratio*; ICC = intraclass correlation coefficient.

**Table 4:** Results of rankFD main and interaction effect, *p*-value with and without correction.

Metrics	Pool of Data	Effect	*p*-Value without Correction	*p*-Value with Correction	ANOVA Type Statistic (ATS)
*UR*	All data	Group	**<0.001**	**<0.001**	33.5
		Task	**<0.001**	**<0.01**	5.5
		Group XTask	0.47	-	-
	Task 3	Group	**<0.01**	0.06	9.7
	Task 5	Group	**<0.001**	**<0.001**	42.4
	Task 6	Group	**<0.01**	**<0.05**	10.9
	Task 3 vs. 5	Group	**<0.001**	**<0.001**	29.5
		Task	**<0.01**	0.08	11.2
		Group X Task	0.89	-	-
	Task 3 vs. 6	Group	**<0.001**	**<0.01**	19.5
		Task	0.66	-	-
		Group XTask	0.72	-	-
	Task 5 vs. 6	Group	**<0.001**	**<0.001**	27.8
		Task	**<0.05**	0.76	5.5
		Group X Task	0.89	-	-
*percentage of dominant use*	All data	Group	**<0.001**	**<0.001**	34.0
		Task	**<0.001**	**<0.001**	10.1
		Group X Task	0.34	-	1.2
	Task 3	Group	**<0.01**	**<0.05**	14.8
	Task 5	Group	**<0.001**	**<0.01**	38.7
	Task 6	Group	**<0.01**	0.14	10.2
	Task 3 vs. 5	Group	**<0.001**	**<0.001**	34.8
		Task	**<0.001**	**<0.01**	23.4
		Group X Task	0.70	-	-
	Task 3 vs 6	Group	**<0.001**	**<0.001**	23.8
		Task	0.28	-	-
		Group XTask	0.95	-	-
	Task 5 vs. 6	Group	**<0.001**	**<0.001**	33.9
		Task	**<0.001**	**<0.001**	35.3
		Group X Task	0.34	-	-
*percentage of non dominant use*	All data	Group	**<0.001**	**<0.001**	26.2
		Task	**<0.05**	0.50	-
		Group X Task	0.24	-	-
	Task2	Group	**<0.001**	**<0.05**	19.8
	Task 3	Group	**<0.05**	0.82	6.5
	Task 5	Group	**<0.05**	1	4.7
	Task 6	Group	**<0.05**	0.62	7.1
	Task 2 vs. 3	Group	**<0.01**	**<0.01**	18.7
		Task	**<0.05**	0.57	6.7
		Group X Task	0.19	-	-
	Task 2 vs 5	Group	**<0.001**	**<0.001**	24.7
		Task	0.52	-	-
		Group X Task	**<0.05**	1	4.4
	Task 2 vs. 6	Group	**<0.001**	**<0.001**	23.6
		Task	0.15	-	-
		Group X Task	0.47	-	-
	Task 3 vs. 5	Group	**<0.01**	0.21	8.8
		Task	**<0.001**	**<0.05**	15.1
		Group X task	0.63	-	-
	Task 3 vs. 6	Group	**<0.01**	0.06	11.9
		Task	0.23	-	-
		Group X task	0.72	-	-
	Task 5 vs. 6	Group	**<0.01**	0.07	11.8
		Task	**0.05**	1	4.1
		Group X Task	0.37	-	-
*percentage of bimanual use*	All data	Group	**<0.01**	**<0.01**	13.9
		Task	**<0.001**	**<0.001**	13.6
		Group X Task	0.75	-	-
	Task 3	Group	**0.05**	1	4.4
	Task 5	Group	**<0.05**	0.22	8.1
	Task 3 vs. 5	Group	**<0.01**	**<0.05**	12.6
		Task	**<0.001**	**<0.001**	45.0
		Group X Task	0.40	-	-

Legend: *UR* = *Use ratio*, only analyses yielding significant results are presented; Task 1 = Cleaning the table; Task 2 = Making coffee; Task 3 = Setting the table; Task 4 = Pouring a glass of water; Task 5 = Folding towels; Task 6 = Putting toothpaste on a toothbrush.

## Data Availability

The data presented in this study are available on request from the corresponding author. The data are not publicly available due to restrictions related to ethical approval.
